# DEAD-Box Helicase 17 Promotes Amyloidogenesis by Regulating BACE1 Translation

**DOI:** 10.3390/brainsci13050745

**Published:** 2023-04-29

**Authors:** Yue Liu, Guifeng Zhou, Li Song, Qixin Wen, Shiqi Xie, Long Chen, Lu Wang, Xiaoyong Xie, Xue Chen, Yalan Pu, Guojun Chen

**Affiliations:** Department of Neurology, The First Affiliated Hospital of Chongqing Medical University, Chongqing Key Laboratory of Neurology, 1 Youyi Road, Chongqing 400016, China

**Keywords:** DDX17, amyloidogenesis, BACE1, Alzheimer’s disease, RNA helicase, APP, β-amyloid protein, translation, 5′UTR, RBP

## Abstract

Amyloidogenesis is one of the key pathophysiological changes in Alzheimer’s disease (AD). Accumulation of the toxic Aβ results from the catalytic processing of β-amyloid precursor protein (APP) associated β-amyloid converting enzyme 1 (BACE1) activity. It is reported that dead-box helicase 17 (DDX17) controls RNA metabolism and is involved in the development of multiple diseases. However, whether DDX17 might play a role in amyloidogenesis has not been documented. In the present study, we found that DDX17 protein level was significantly increased in HEK and SH-SY5Y cells that stably express full-length APP (HEK-APP and Y5Y-APP) and in the brain of APP/PS1 mice, an animal model of AD. DDX17 knockdown, as opposed to DDX17 overexpression, markedly reduced the protein levels of BACE1 and the β-amyloid peptide (Aβ) in Y5Y-APP cells. We further found that DDX17-mediated enhancement of BACE1 was selectively attenuated by translation inhibitors. Specifically, DDX17 selectively interacted with the 5′ untranslated region (5′UTR) of BACE1 mRNA, and deletion of the 5′UTR abolished the effect of DDX17 on luciferase activity or protein level of BACE1. Here, we show that the enhanced expression of DDX17 in AD was associated with amyloidogenesis; through the 5′UTR-dependent BACE1 translation, DDX17 might serve as an important mediator contributing to the progression of AD.

## 1. Introduction

Alzheimer’s disease (AD) is the most common neurodegenerative disease affecting more than 35 million people worldwide [1], with a particular onset and course of cognitive decline associated with age [2]. Anatomical studies reveal that atrophy of the frontal, temporal and parietal cortices and hippocampus develops along with different disease stages [3]. Despite that oxidative stress and inflammation play an important role in cognitive impairment [4], the pathology of AD is characterized by β-amyloid plaque (Aβ) deposition and neurofibrillary tangles (NFTs) of hyperphosphorylated tau [5,6]. Importantly, Aβ remains one of the diagnostic biomarkers that distinguish AD from other dementias [7,8].

Amyloidogenesis refers to overproduction or the inhibited clearance of Aβ, leading to an excessive Aβ accumulation. It is suggested that Aβ is generated from the only source of amyloid precursor protein (APP), which can be regulated by the amyloid and non-amyloid pathways. The former involves the enzymatic activity of β-amyloid converting enzyme 1 (BACE1) [9], whereas the latter is associated with the α-secretase, a disintegrin and metalloproteinase 10 (ADAM10) [10]. Previous studies demonstrate that protein level and activity of BACE1 are increased in the brain of AD, and generations of the improved BACE1 inhibitors have shown potential hope for the treatment [11,12]. Moreover, BACE1 expression is regulated by a variety of factors including oxidative stress, inflammation, and calcium overload, indicating that the upstream signaling of BACE1 is closely associated with the pathogenesis of AD [13,14]. Thus, it is important to understand how BACE1 links the key molecular alterations with amyloidogenesis.

DEAD-box helicase 17 (DDX17) belongs to the DEAD-box family of ATP-dependent RNA helicase [15]. These proteins are characterized by the conserved motif Asp-Glu-Ala-Asp [16] and are essential for RNA-protein complex dynamics, RNA decay, and translation [17,18]. It is reported that DDX17 is an RNA binding protein (RBP) that recognizes specific RNA structure and promotes miRNA processing [19], and serves as an essential mediator of sterile NLRC4 inflammasome activation by regulating retrotransposon RNAs [20,21]. DDX17 is also involved in DNA damage repair and cancer development [22,23,24]. In particular, DDX17 affects the pathological progress of cancer through a variety of mechanisms, such as formation of the microprocessor complex, transcriptional regulation, and RNA binding [25,26]. Moreover, the role of DDX17 in the alternative splicing of many important tumor-related genes has also been reported [27,28]. In neurological diseases, DDX17 is involved in the pathology of amyotrophic lateral sclerosis, an ophthalmoplegic subphenotype of myasthenia gravis and glioma [22,29,30]. However, whether DDX17 might be involved in AD is currently uncertain.

In this study, we aim to explore whether DDX17 participates in AD pathology by influencing amyloidogenesis. An elevated level of DDX17 is found in the brain of APP/PS1 mice. We further define that DDX17 preferentially affects the amyloid pathway by regulating BACE1 translation, through binding to the 5′ untranslated region (5′UTR) of BACE1 mRNA. The close association of DDX17 with amyloidogenesis suggests that DDX17 could serve as a new potential target for the treatment of AD.

## 2. Materials and Methods

DDX17 protein level was first assessed in cellular and animal models of AD, followed by evaluation of protein expression levels of APP, BACE1, ADAM10, and amyloidogenesis by DDX17 knockdown or overexpression. Through pharmacological methods, the potential mechanisms of DDX17-induced alteration of BACE1 expression were further determined, and DDX17 binding to the 5′UTR of BACE1 mRNA in association with the 5′UTR activity was assessed by RNA pulldown and luciferase reporter assay [31,32].

### 2.1. Animal Model

APPswe/PS1E9 (APP/PS1, RRID: MMRRC_034829-JAX) [33] transgenic mice were obtained from Ensiweier (Chongqing, China). Controls were generated from littermates without AD phenotype. All the animals were fed freely in the Experimental Animal Center of Chongqing Medical University in cycles of 12 h dark/12 h light, and experimental protocols strictly abided by the management of the Ethics Commission of Chongqing Medical University in accordance with international standards. Brain tissue samples were obtained from male mice at 9 months. After anesthesia by pentobarbital (100 mg/kg, i.p), brain tissues were quickly collected and stored in liquid nitrogen.

### 2.2. Antibodies and Reagents

The following antibodies were purchased from Abcam (Cambridge, United Kingdom): ADAM10 (ab1997, 1:1000), DDX17 (ab180190, 1:1000), and BACE1 (ab2077, 1:1000). Antibodies against APP and CTFs (A8717, 1:1000) were purchased from Sigma-Aldrich (St. Louis, MO, USA), and those against GADPH (60004-1-Ig, 1:10,000) were obtained from Proteintech (Wuhan, China).

### 2.3. Cell Lines and Chemicals

HEK293 (human embryonic kidney) cell lines and SH-SY5Y (human neuroblastoma) cell lines were obtained from the Type Culture Collection of the Chinese Academy of Sciences (Shanghai, China). HEK293 were maintained in DMEM (Gibco, Rockville, MD, USA) with 10% of fetal bovine serum (FBS, Hyclone). SH-SY5Y(Y5Y) were cultured in F-12 (Gibco, Rockville, MD, USA) supplemented with 10% of FBS. HEK-APP was generated from HEK293 stably expressing human full-length APP695 as previously described [34]. HEK-APP cells were cultured in DMEM supplemented with 10% of FBS and 200 mg/mL of G418 (Sigma-Aldrich, St. Louis, MO, USA). Y5Y-APP (SH-SY5Y cells stably expressing human full-length APP695) cells were cultured in F-12 with 10% of FBS and 200 mg/mL of G418 [35]. All cells were cultured in a humidified incubator at 37 °C and 5% CO_2_.

The following chemicals were purchased from Sigma-Aldrich (St. Louis, MO, USA): lysosomal inhibitor chloroquine (CQ), protease inhibitor MG132, the transcriptional inhibitor actinomycin D (ActD), and the protein biosynthesis inhibitor cycloheximide (CHX). The eIF4E/eIF4G interaction inhibitor 4EGI-1 were from Selleck (Houston, TX, USA). 4EGI-1, MG132, ActD, and CHX were all dissolved in dimethyl sulfoxide (DMSO). The final concentration of DMSO was at least 1:2000. CQ was dissolved in sterilized double-distilled H_2_O.

### 2.4. Plasmid and Transfection

Human DDX17 pcDNA3.1(+) plasmid, control vector pcDNA3.1(+), human BACE1 +5′UTR (containing the 5′UTR and the entire human BACE1 coding region) pcDNA3.1(+), and human BACE1-5′UTR (only containing the entire human BACE1 coding sequence) pcDNA3.1(+) were obtained from YouBio (Changsha, China). The BACE1 +5′UTR-pmirGLO and BACE1 −5′UTR-pmirGLO were from Sangon Biotech (Shanghai, China). The siRNA oligonucleotide sequences for DDX17 (SiDDX17) and non-targeting control (NC) were designed by GenePharma Biotech (Shanghai, China) as follows: SiDDX17 (sense: GCUGCUUAUGGCACCAGUAGCUAUA; antisense: UAUAGCUACUGGUGCCAUAAGCAGC); and NC (sense: UUCUCCGAACGUGUCACGUTT; antisense: ACGUGACACGUUCGGAGAATT). Cells were transfected with Lipofectamine^TM^ 3000 (Invitrogen, USA) or Lipofectamine^TM^ RNAiMAX Transfection Reagent (Invitrogen, Carlsbad, CA, USA) mixed with Opti-MEM^TM^ Reduced Serum Media (Gibco, Rockville, MD, USA) according to the manufacturer’s protocols.

### 2.5. Western Blotting

Animal brain tissues or cells were homogenized in ice-cold RIPA buffer (Beyotime, Shanghai, China) supplemented with protease inhibitor cocktail (MedChemExpres, NJ, USA) [35]. All protein samples were ultrasonicated on ice for 30 min and centrifuged at 16,000× *g* for 20 min at 4 °C. BCA Protein Assay Kit (Beyotime, Shanghai, China) was used to detect the concentration of all protein samples. Denatured proteins were separated by 8% SDS-PAGE gels or 16.5% Tris-Tricine-SDS-PAGE gels specific for CTF detection [36]. The PVDF membranes were visualized using electrochemiluminescence (ECL) reagent (Beyotime, Shanghai, China) and analyzed with the Fusion FX5 image system (Vilber Lourmat, Marne-la-Vallee, France). The protein expression was analyzed by ImageJ and normalized to the amount of GADPH.

### 2.6. Luciferase Activity Assay

The Dual-Lumi II Luciferase Reporter Gene Assay Kit (Beyotime, Shanghai, China) was used in this experiment. Y5Y-APP cells were seeded in a 96-well plate for 24 h before transfection. After 48 h of transfection, luciferase activity was measured by a GloMax microplate luminometer (Promega, Madison, WI, USA) according to the instructions of the manufacturer [35].

### 2.7. RNA Pulldown Assay

RiboTrap Kit (MBL, Tokyo, Japan) was used to perform the RNA pulldown assay according to the manufacturer’s instructions. Briefly, using the linearized BACE1 5′UTR pcDNA3.1(+) plasmid as a template, the 5-bromo-UTP (BrU) was incorporated into the BACE1 5′UTR randomly through in vitro transcription (Riboprobe in vitro Transcription Systems-T7, Promega). Then, the anti-BrdU antibody was conjugated to protein G beads (Pierce), and the cytoplasmic extracts from cell lysates were incubated with the BrU-labeled BACE1 5′UTR and the antibody-conjugated beads for 2 h at 4 °C [31]. After washing and eluting, the samples were subjected to Western blotting.

### 2.8. Enzyme-Linked Immunosorbent Assay (ELISA)

The human Aβ40 and Aβ42 levels in Y5Y-APP cells were detected by ELISA as previously described [37]. In brief, the culture medium of Y5Y-APP cells was collected and then centrifuged at 4 °C for 30 min at 14,000 rpm. The expression levels Aβ40 and Aβ42 were, respectively measured by the commercially available ELISA kits according to the manufacturer’s specification (Elabscience, E-EL-H0542c for Aβ40, and E-EL-H0543c for Aβ42, Wuhan, China).

### 2.9. Statistical Analysis

All data were obtained from at least three independent replicate experiments and were presented as mean ± standard deviation (SD). Data were statistically analyzed by GraphPad Prism version 8.0 (GraphPad Software, La Jolla, CA, USA). Comparison between two groups was analyzed by unpaired Student’s *t*-test. The differences were considered to be statistically significant when *p* < 0.05.

## 3. Results

### 3.1. DDX17 Protein Levels Were Increased in Cellular and Animal Models of AD

**Figure 1:**
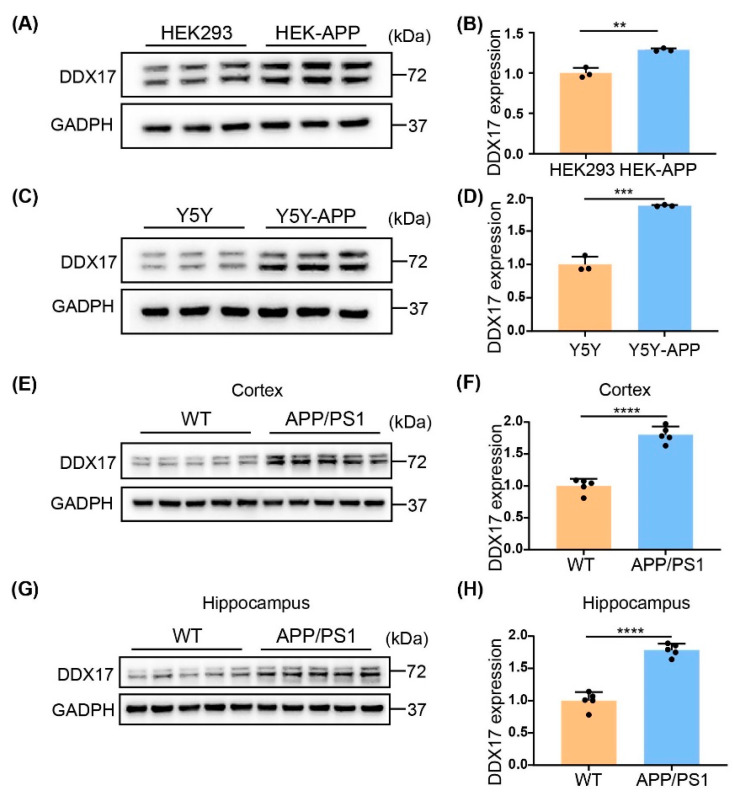
DDX17 protein levels are increased in cellular and animal models of AD. (**A**,**B**), Representative Western blots (**A**) and quantitative analysis (**B**) of the protein expression levels of DDX17 in HEK-293 and HEK-APP cells. (**C**,**D**) Representative Western blots (**C**) and quantitative analysis (**D**) of the protein expression levels of DDX17 in Y5Y and Y5Y-APP cells. (**E**,**F**) Representative Western blots (**E**) and quantitative analysis (**F**) of the protein expression levels of DDX17 in the cortex of wild-type (WT) and APP/PS1 mice at 9 months of age (n = 5 in each group). (**G**,**H**) Representative Western blots (**E**) and quantitative analysis (**F**) of the protein expression levels of DDX17 in the hippocampus of WT and APP/PS1 mice at 9 months of age (n = 5 in each group). Data are expressed as means ± SD. ** *p* < 0.01, *** *p* < 0.001, **** *p* < 0.0001.

DDX17 has two major mRNA isoforms responsible for two protein isomers, respectively: p72 and p82, which are commonly co-expressed in cell lines and tissues without functional difference [24]. To determine whether the expression of DDX17 is associated with AD, we first examined the protein levels of DDX17 in HEK-APP and SH-SY5Y-APP (Y5Y-APP) cells, which could serve as a cellular model of AD because of the higher Aβ levels as a result of APP overexpression [38]. As shown in Figure 1A–D, the protein expression level of DDX17 was significantly increased in HEK-APP and Y5Y-APP cells compared with the corresponding cells in which APP was not stably overexpressed. Similarly, in both the cortex and hippocampus of the APP/PS1 mice, a significantly higher DDX17 protein level was found relative to age-matched controls (Figure 1E–H). These results indicated that DDX17 protein levels were increased in cellular and animal models of AD.

### 3.2. DDX17 Regulated APP Processing by Enhancing BACE1 Protein Level

**Figure 2:**
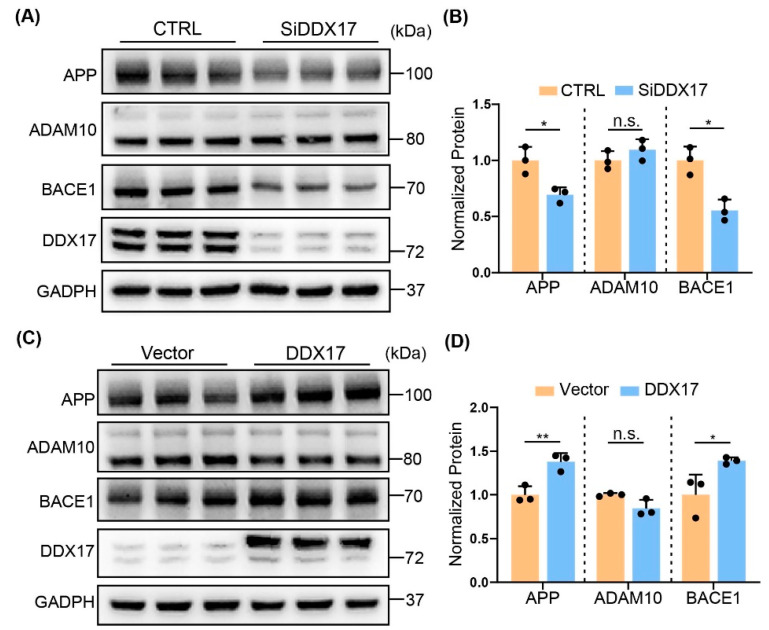
DDX17 regulates APP processing by increasing BACE1 protein levels. (**A**,**B**) Representative Western blots (**A**) and quantitative analysis (**B**) of APP, ADAM10, and BACE1 in Y5Y-APP cells. Samples were collected after transfections with control (CTRL) or DDX17 siRNA (SiDDX17) for 48 h. (**C**,**D**) Representative Western blots (**C**) and quantitative analysis (**D**) of APP, ADAM10, and BACE1 in Y5Y-APP cells. Samples were collected after transfections with vector or DDX17 for 48 h. All values were normalized to control or vector. Data are expressed as means ± SD; n.s. indicates no significant difference. * *p* < 0.05, ** *p* < 0.01.

The altered DDX17 protein expression levels prompted us to speculate that DDX17 might be involved in amyloidogenesis. Whereas BACE1 is responsible for Aβ and β-CTF generation by APP processing [39,40], the α-secretase a disintegrin and metalloproteinase 10 (ADAM10) facilitates the non-amyloid cleavage of APP, leading to a decreased level of Aβ [41]. Thus, we assessed the protein expression levels of APP, BACE1, and ADAM10 in Y5Y-APP cells transiently transfected with DDX17 siRNA. As shown in Figure 2A,B, knockdown of DDX17 significantly decreased the protein levels of APP and BACE1, while those of ADAM10 were not obviously altered. In contrast, DDX17 overexpression significantly increased the protein levels of APP and BACE1, without altering those of ADAM10 (Figure 2C,D). These results indicated that the protein expression levels of BACE1 were preferentially affected by DDX17.

### 3.3. DDX17 Regulated Amyloidogenesis

**Figure 3:**
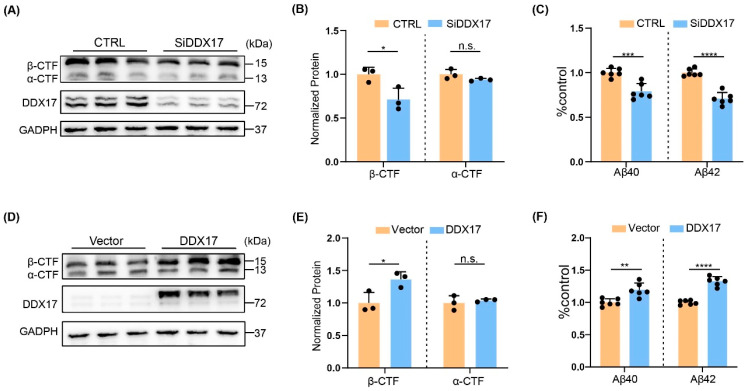
DDX17 regulates the protein levels of β-CTF and Aβ. (**A**,**B**) Representative Western blots (**A**) and quantitative analysis (**B**) of β-CTF and α-CTF in Y5Y-APP cells. Samples were collected after transfections with control (CTRL) or DDX17 siRNA (SiDDX17) for 48 h. (**C**), Protein expression levels of Aβ40 and Aβ42 in culture medium from Y5Y-APP cells transfected with SiDDX17 relative to CTRL. (**D**,**E**) Representative Western blots (**D**) and quantitative analysis (**E**) of β-CTF and α-CTF in Y5Y-APP cells. Samples were collected after transfections with vector or DDX17 for 48 h. (**F**) Protein expression levels of Aβ40 and Aβ42 in culture medium from Y5Y-APP cells transfected with DDX17 relative to vector. All data are expressed as means ± SD; n.s. indicates no significant difference. * *p* < 0.05, ** *p* < 0.01, *** *p* < 0.001, **** *p* < 0.0001.

To determine whether the altered BACE1 expression by DDX17 was associated with amyloidogenesis, we assessed the protein expression levels of the metabolites of APP including α/β-CTFs and Aβ [42] in Y5Y-APP cells. Cells were treated with the γ-secretase inhibitor DAPT for better detection of CTFs after transfection with DDX17 siRNA [43]. First, we found that DDX17 silencing significantly reduced the protein levels of β-CTF but not α-CTF (Figure 3A,B). Accordingly, the expression levels of Aβ40 and Aβ42 were significantly decreased (Figure 3C). Moreover, an opposite effect was observed in Y5Y-APP cells overexpressing DDX17, in which the protein levels of β-CTF and Aβ40/42 were significantly increased without altering those of α-CTF (Figure 3D–F). These results indicated that DDX17 controlled amyloidogenesis that was associated with BACE1 protein level and activity. 

### 3.4. DDX17-Mediated Regulation of BACE1 Involved a Translational Mechanism

**Figure 4:**
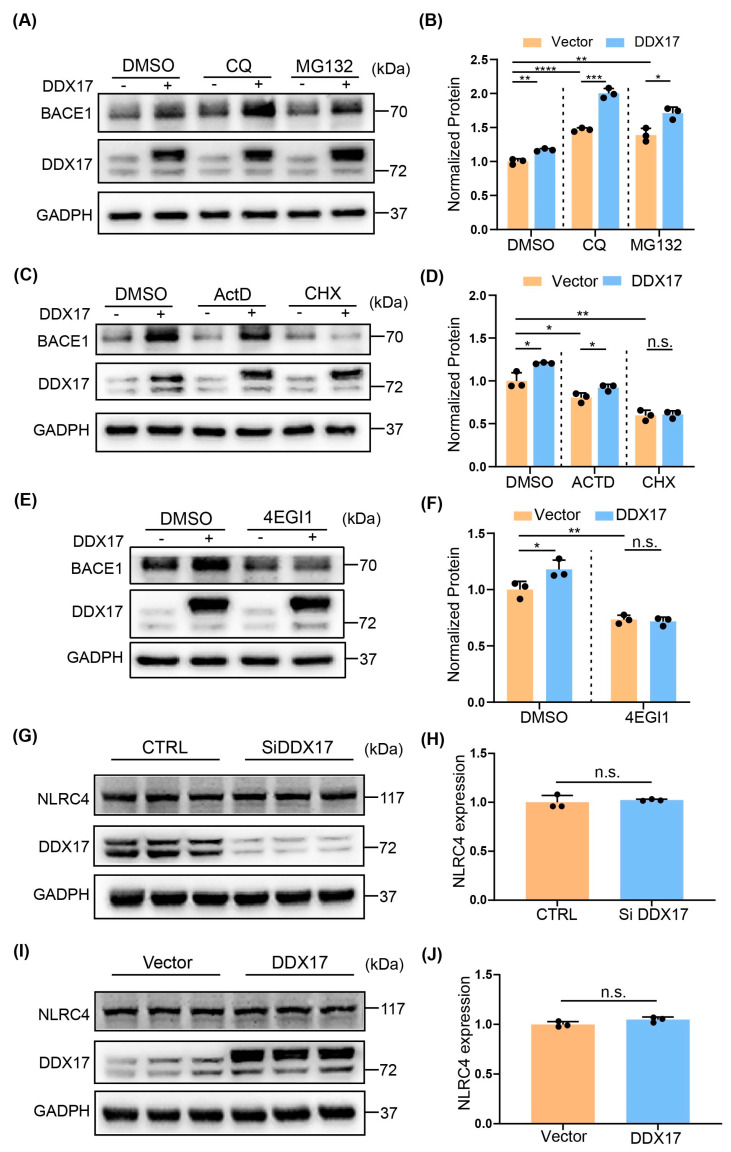
DDX17−mediated regulation of BACE1 involves a translational mechanism. (**A**,**B**) Representative Western blots (**A**) and quantitative analysis (**B**) of BACE1 protein expression levels in Y5Y-APP cells. Cells were transfected with vector or DDX17 and further treated with or without CQ (100 μM for 6 h) or MG132 (1 μM for 6 h). (**C**,**D**) Representative Western blots (**C**) and quantitative analysis (**D**) of BACE1 levels in Y5Y-APP cells. Cells were transfected with vector or DDX17 and further treated with or without ActD (0.1 μM for 12 h) or CHX (5 μM for 6 h). (**E**,**F**) Representative Western blots (**E**) and quantitative analysis (**F**) of BACE1 levels in Y5Y-APP cells. Cells were transfected with vector or DDX17 and further treated with or without 4EGI1(25 μM for 6 h). (**G**,**H**) Representative Western blots (**G**) and quantitative analysis (**H**) of NLRC4 expression levels in Y5Y-APP cells with DDX17 knockdown. (**I**,**J**) Representative Western blots (**I**) and quantitative analysis (**J**) of NLRC4 expression levels in Y5Y-APP cells overexpressing DDX17. Data are expressed as means ± SD; n.s. indicates no significant difference. * *p* < 0.05, ** *p* < 0.01, *** *p* < 0.001, **** *p* < 0.0001.

Previous studies have shown that the expression of BACE1 can be regulated at several levels, including gene transcription, translation, and protein degradation [9,44,45]. Thus, we first investigated whether DDX17-mediated regulation of BACE1 was regulated at the protein degradation level. Y5Y-APP cells were transfected with vector or DDX17 and then treated with lysosome inhibitor chloroquine (CQ) or proteasome inhibitor MG132 [46]. We found that although CQ or MG132 increased the basal protein level of BACE1 significantly, neither CQ nor MG132 prevented the up-regulation of BACE1 (Figure 4A,B). Next, we examined whether DDX17-mediated upregulation of BACE1 was regulated at the transcriptional or translational level. We used the transcription inhibitor ActD and the translation inhibitor CHX for our study [47]. As shown in Figure 4C,D, whereas the basal protein levels of BACE1 were significantly decreased in cells treated with ActD or CHX, DDX17 overexpression-induced enhancement of BACE1 protein was blocked by CHX but not ActD. This result suggested that a translation mechanism is involved in DDX17-mediated BACE1 regulation. Then, we used 4EGI-1, a small molecule inhibitor of cap-dependent translation for validation [48,49,50]. As shown in Figure 4E,F, 4EGI-1 significantly reduced the basal level of BACE1 and further blocked the effect of DDX17 on BACE1. It is reported that DDX17 is implicated in inflammation by mediating NLRC4 inflammasome activation [21], suggesting that NLRC4 in association with inflammation could be responsible for the elevated level of BACE1 [13]. As shown in Figure 4G–J, the protein level of NLRC4 was not significantly altered by DDX17 knockdown or overexpression. These results indicated that, without altering the level of NLRC4, DDX17-mediated upregulation of BACE1 involved a translational mechanism.

### 3.5. The Effect of DDX17 on BACE1 Translation Was Dependent on the 5′UTR

**Figure 5:**
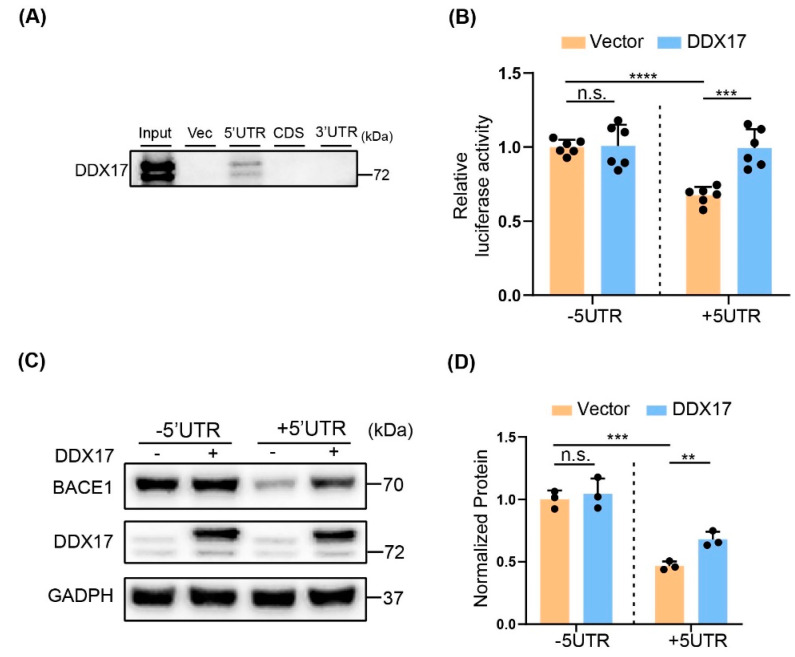
DDX17 regulates BACE1 translation through the 5′UTR. (**A**) Representative Western blots of DDX17 in immunoprecipitated extracts by RNA pulldown using BrU−labelling of the 5′UTR, coding sequence (CDS) and 3′UTR of BACE1 mRNA. (**B**) Relative luciferase activities in Y5Y-APP cells. Cells were transiently co-transfected with vector or DDX17 plasmid, and human BACE1 mRNA construct either deleting (−5′UTR) or containing the 5′UTR (+5′UTR). (**C**,**D**) Representative Western blots (**C**) and quantitative analysis (**D**) of BACE1 protein expression levels in Y5Y-APP cells. Cells were transiently co-transfected with vector or DDX17 plasmid and human BACE1 mRNA in which the 5′UTR was either deleted (−5′UTR) or included (+5′UTR). n.s. indicates no significant difference. ** *p* < 0.01, *** *p* < 0.001, **** *p* < 0.0001.

The helicase activity suggests that function of DDX17 could be associated with translation [51]. Thus, we assessed the potential interaction between DDX17 and different fragments of BACE1 mRNA, including the 5′UTR, coding sequence (CDS), and the 3′UTR. As shown in Figure 5A, RNA-pulldown assay revealed that DDX17 selectively bound to the 5′UTR, which is known to be critical for BACE1 translation initiation [45,52,53]. Accordingly, the luciferase activity was significantly increased by DDX17 overexpression in cells co-transfected with BACE1 +5′UTR, but not with BACE1 −5′UTR construct, in which the 5′UTR was deleted (Figure 5B). To validate that DDX17-mediated upregulation of BACE1 protein levels was also dependent on the 5′UTR, we detected the protein expression level of BACE1 in Y5Y-APP cells co-transfected with DDX17 and BACE1 constructs containing (+5′UTR) or deleting (−5′UTR) the 5′UTR. We found that the basal protein level of BACE1 was dramatically higher in the Y5Y-APP cells overexpressing the −5′UTR relative to +5′UTR, which was in line with previous reports showing that the 5′UTR inhibits BACE1 translation [32,54]. In addition, the DDX17-mediated enhancement of BACE1 was presented in +5′UTR but not −5′UTR. These data collectively indicated that DDX17 promoted BACE1 translation through the 5′UTR-dependent mechanism.

Altogether, our data indicated that an increased protein level of DDX17 was found in both cellular and animal models of AD. The role of DDX17 in amyloidogenesis was demonstrated by that DDX17 overexpression enhanced BACE1 translation in the 5UTR-dependent manner, whereas DDX17 knockdown exerted an opposite effect.

## 4. Discussion

The present study reveals that the protein level of DDX17 is significantly elevated in both cellular and animal models of AD. In line with this, the mRNA level of DDX17 is significantly increased in the selected area of AD patients (http://www.alzdata.org, accessed on 12 March 2022) [55]. We also show that DDX17 enhances BACE1 translation through the 5′UTR of mRNA, leading to an increased BACE1 protein level and Aβ generation. 

The exact pathophysiological mechanisms of AD remain incompletely understood. In recent years, accumulating evidences have suggested that alterations in RNA processing and RBPs are involved in AD [56]. For example, the level of RNA splicing, along with small ribonuclear protein complex, is changed in AD patients [57]. Mitochondrial and cytosolic tRNA methylation are dysregulated in animal model of AD [58]. Using multiple approaches, including RNAseq and proteomics, RNA-binding protein ELAVL4 is identified to attenuate the molecular alterations of AD [59]. As DDX17 is one of the most characterized RBPs [60], the current study provides a link between RBP and the amyloidogenesis, which should shed new light on the understanding of disease progression. 

The DEAD-box RNA helicases are the largest family of RNA helicases. They play an important role in gene expression and RNA metabolism ranging from pre-mRNA splicing, mRNA export and decay and translation initiation, to ribosome biogenesis [61,62]. DDX17, in particular, is involved in various types of human diseases [24,25,29]. For instance, DDX17 regulates breast cancer pathology by promoting the transcription of NFAT5 target genes [63], and facilitates hepatocellular carcinoma metastasis by regulating alternative splicing, leading to the production of oncogenic RNA subtypes [64]. It is reported that upregulation of DDX17 is associated with the progression and poor prognosis in glioma [29], in addition to an enhancement of the gefitinib resistance via activation of β-catenin [65]. To the best of our knowledge, the role of DDX17 in BACE1 regulation in association with the pathology of AD, has not been previously reported.

Aβ slowly and progressively accumulates in the brain long before the presence of clinical symptoms [66]. Aβ pathogenesis starts with altered cleavage of APP which is an integral protein on the plasma membrane [67]. In the catalytic processing of APP, BACE1 functions as a rate-limiting enzyme that links to amyloidogenesis [68]. Importantly, BACE1 cross-talks with cellular and molecular mechanisms that are in close association with the pathophysiology of AD [13]. In our study, we provide evidence that DDX17 controls amyloidogenesis by selectively regulating protein levels BACE1 without altering those of ADAM10. A translational mechanism in BACE1 regulation by DDX17 is supported by that the translation inhibitor CHX or 4EGI-1 [48,69], but not the lysosome inhibitor CQ, the proteasome inhibitor MG132, or the transcription inhibitor ActD, prevents DDX17-mediated regulation of BACE1. Thus, the translational control of BACE1 links DDX17 to amyloidogenesis. It is worth noting that DDX17 is implicated in inflammation, which is mainly mediated by its RNA-binding activity and could be associated with NFκB [21,70]. This raises the possibility that DDX17 could regulate BACE1 expression through inflammation. Previous studies demonstrate that inflammation, oxidative stress, and NFκB promote BACE1 expression through a transcriptional mechanism [13,71,72]. In our study, the level of inflammasome maker NLRC4 that is not altered by DDX17 knockdown or overexpression and the failure of transcription inhibitor ActD to prevent DDX17-induced BACE1 expression suggest that inflammation might not play a direct role.

It is reported that the 5′UTR is critical for BACE1 translation and can be regulated by the eukaryotic initiation factors and translation elongation factor Tu of mitochondria [46,73,74]. In our study, the interaction between DDX17 and the 5′UTR is demonstrated by RNA pull-down assay. Moreover, the functional role of DDX17 in direct regulation of the 5′UTR is supported by that the enhanced luciferase activity and protein level of BACE1 occur only in the presence of 5′UTR, indicating that DDX17 acts as a key RBP in BACE1 translation initiation. 

Study limitation: the present study focuses only on amyloidogenesis by DDX17, without investigating changes in glial fibrillary acidic protein and Tau in association with phosphorylation, which are considered as potential biomarkers for AD [75]. Given that RBPs including DDX17, are the major components of Tau interactors [76], it could be possible that DDX17 and RBP-enriched stress granules might be critical for Tau phosphorylation and neurofibrillary tangles [77]. Therefore, the role of DDX17 in Tau regulation may deserve a separate and independent study in the future.

## 5. Conclusions

Although alteration of RBPs has been reported as one of the prominent features of AD, how RBPs might be involved in amyloidogenesis remains an open question. Our study provides evidence that DDX17 functions as RBP that controls BACE1 translation through binding to the 5′UTR. Given that RBPs are essential for RNA processing and cellular functions, our study highlights a potential link of DDX17 with the pathophysiology of AD.

## Data Availability

All data are available in the main text.
